# Environmental and Patient Impact of Applying a Point-of-Care Ultrasound Model in Primary Care: Rural vs. Urban Centres

**DOI:** 10.3390/ijerph17093333

**Published:** 2020-05-11

**Authors:** Francesc X Marín-Gomez, Jacobo Mendioroz Peña, Vicenç Canal Casals, Marcos Romero Mendez, Ana Darnés Surroca, Antoni Nieto Maclino, Josep Vidal-Alaball

**Affiliations:** 1 Health Promotion in Rural Areas Research Group, Institut Català de la Salut, 08272 Sant Fruitós de Bages, Spain; jmendioroz.cc.ics@gencat.cat (J.M.P.); jvidal.cc.ics@gencat.cat (J.V.-A.); 2 Unitat de Suport a la Recerca de la Catalunya Central, Fundació Institut Universitari per a la Recerca a l’Atenció Primària de Salut Jordi Gol i Gurina, 08007 Barcelona, Spain; 3 Servei d’Atenció Primària Osona, Gerència Territorial de Barcelona, Institut Català de la Salut, 08500 Vic, Barcelona, Spain; 4 Centre d’Atenció Primària Vic Nord, Gerència Territorial de Barcelona, Institut Català de la Salut, 08500 Vic, Barcelona, Spain; vcanal.cc.ics@gencat.cat; 5 Centre d’Atenció Primària St. Quirze de Besora, Gerència Territorial de Barcelona, Institut Català de la Salut, 08580 Sant Quirze de Besora, Barcelona, Spain; mromerom.cc.ics@gencat.cat; 6 Centre d’Atenció Primària Manlleu, Gerència Territorial de Barcelona, Institut Català de la Salut, 08560 Manlleu, Barcelona, Spain; adarnes.cc.ics@gencat.cat; 7 Centre d’Atenció Primària Sta. Eugènia de Berga, Gerència Territorial de Barcelona, Institut Català de la Salut, 08507 Santa Eugènia de Berga, Barcelona, Spain; anieto.cc.ics@gencat.cat

**Keywords:** point-of-care systems, ultrasonography, traffic-related pollution, primary care

## Abstract

Motor vehicles are a major contributor to air pollution, and the exposure to this human-caused air pollution can lead to harmful health effects. This study evaluates the impact of the provision of point-of-care ultrasounds (POCUS) by primary care (PC) to avoid the patient’s need to travel to a specialized service. The study estimates the costs and air pollution avoided during 2019. The results confirm that performing this ultrasound at the point of care reduces the emission of 61.4 gr of carbon monoxide, 14.8 gr of nitric oxide and 2.7 gr of sulfur dioxide on each trip. During the study, an average of 17.8 km, 21.4 min per trip and almost 2000 L of fuel consumed in a year were avoided. Performing POCUS from PC reduces fuel consumption and the emission of air pollutants and also saves time and money. Furthermore, only 0.3% of the scans had to be repeated by radiologists. However, more studies with more participants need to be done to calculate the exact impact that these pollution reductions will have on human health.

## 1. Introduction

Over the last decade, numerous studies have shown that exposure to the current levels of human-caused air pollution can lead to a wide range of harmful health effects [1]. Current data makes it possible to give a rough estimate as to the number of health problems which can be directly attributed to air pollution in each territory and to detect the avoidable sources of such pollution. An assessment of the risks associated with human activities is an important tool for quantifying the current problem in terms of its size and how it is evolving, prior to being able to overcome such problems. Recent research shows that motor vehicles are a major contributor to air pollution [2] and suggest that harmful effects exist even at very low levels, with no clear evidence that there is a threshold below which pollution has no effects on people’s health [3,4].

Several studies have analysed the environmental impact of the provision by primary care centres (PCCs) of various healthcare services, which up until now have been provided by hospitals or specialized services [5,6], thus avoiding the patient’s need to travel to a specialized service [6,7,8]. However, the potential impact of conducting ultrasound scans in PCCs with regard to pollutant emissions has not previously been studied. At a time of growing interest in reducing the environmental impact of health-related activities [9], this study assesses the impact of conducting ultrasound scans in PCCs as a means to reduce the environmental footprint of this process. Nevertheless, the need for a medical test, such as an ultrasound, can be affected by geographical barriers, and often the distance which users need to travel can cause particular problems, especially for patients in rural communities or places where there is an insufficient number of doctors or deficiencies in the provision of health services [10,11].

Given these circumstances, in recent years ultrasound equipment has been introduced in PCCs to be used by a group of family medicine professionals with special training, fostering a programme of continuous training and an increasing volume of activity. For several years, Catalonia, alongside other autonomous communities, has been committed to equipping healthcare centres with ultrasound scanners and training their staff in how to use them. This study examines the model applied in the Central region of Catalonia, in Osona county, where for some years now [12] ultrasound equipment has been gradually introduced into primary care (PC), beginning with the centres which were the most willing to use them. During this time, the staff has received training in its usage. 

This new ultrasound scan service means patients can visit their general practitioner (GP) to have their test done instead of having to go to the nearest radiology service in the county and waiting for an appointment. Although patients in urban areas live quite close to their referral hospital’s radiology service, this type of service is especially valuable in rural areas where patients find it more difficult to reach the hospital [13]. As a result, the various professional bodies and groups which represent GPs support the use of ultrasound scans in a large number of clinical situations which form part of routine PC since it increases their ability to diagnose and treat cases, optimizing the use of referrals for diagnostic tests and reducing waiting times [14]. Furthermore, having such tests carried out in PC is well received by both users and healthcare professionals [15]. Nevertheless, few studies have been published which study this phenomenon in rural areas [16] and/or in PC [12], despite many scientific organisations offering training in this field.

In spite of the fact that the carrying out of ultrasound scans in PCCs may be seen as a novelty [17], Hahn et al. published a study examining the education and training received by family physicians more than 30 years ago [18], demonstrating that such programmes were cost-effective and provided quality care [19].

The potential for non-radiologists to perform ultrasound scans, what is known as the point-of-care ultrasound (POCUS) model [8], means the technique can actually form part of the consultation, giving it great potential for a timely evaluation and a speedy diagnosis. This in turn makes it highly appealing to GPs, who have increasingly undergone the necessary training to provide this service. The use of ultrasound scans at the patient’s bedside is providing a speedy service to thousands of users [16], avoiding unnecessary travel to specialized services, while representing potential economic savings (environmentally beneficial) for each face-to-face consultation avoided.

Local air pollution and global climate change policies should work together to maximize the benefits of lowering air pollution levels. Evidence suggests that more in-depth cross-city studies have the potential to highlight best practices in both local and global terms [2]. Moreover, as a mobile business, the healthcare sector consumes countless liters of fossil fuels when patients and medical professionals travel to and from their appointments, to pick up prescriptions, or collect test results [20]. The healthcare sector, which has a special focus on health promotion, can thus reasonably be expected to have a moral obligation to set a good example. Ostrom argues that local initiatives are indeed the ones that have the greatest global impact [21]. Hence, potential local mitigation strategies relevant to the health sector are potentially transferable to other countries. 

This study focused on the relatively unexplored use of POCUS as a health sector climate change mitigation and adaptation strategy, evaluating its different uses in rural and urban environments. Overall, this is an example of the growing concern of primary care centers with air pollution affecting their local communities.

## 2. Materials and Methods

This is a retrospective study using administrative data of patients who underwent an ultrasound examination in 2019 by one of the eight GPs of the five primary care centres (PCCs) belonging to the Catalan Institute of Health in Osona county. Of these five centres, two were urban and three rural.

**Figure 1:**
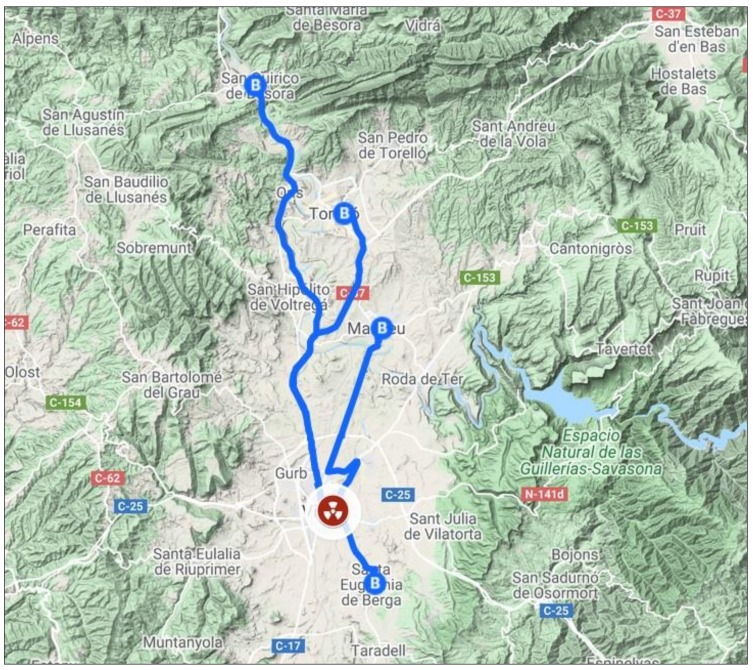
Map showing the primary care centres and their associated diagnostic imaging service in Osona.

The radiology referral service for all of the patients in the PCCs involved in the study was located in the capital of the region, Vic (Figure 1).

This study employed the Primary Care Information System’s [22] criteria to differentiate between urban and rural centres. It defines as rural those PCCs which have an assigned population density of less than 150 inhabitants/km^2^ and less than 10,000 inhabitants [23].

Using the data regarding ultrasound scans, their impact on the journeys undertaken by users in their own vehicles was estimated in terms of the associated costs and the resulting reduction of air pollution. Distances avoided or not travelled were calculated in terms of return journeys from the PCC to the radiology referral service.

The reduction in the emissions of air pollution and greenhouse gases was calculated by multiplying the kilometres avoided by the corresponding emissions of each pollutant. The economic costs saved were derived from calculating the difference between the cost of traveling to the radiology referral service and that of traveling to the nearest PCC. Finally, the time saved was defined as the sum of the duration of the return journey to the radiology referral service. Data from Google Maps was used in order to calculate the distances travelled and the time saved on the journey using the existing road network. The search option “fastest route with the usual traffic” was employed in all instances. To calculate fuel consumption we used the average cost and consumption of a small family car, with fewer than three passengers with no luggage or additional luggage systems, being driven smoothly with an average fuel consumption of 6.9 L/km. The cost of fuel was calculated by averaging the cost of a liter of Gasoline SP95, Gasoline SP98, Diesel A, Diesel A+ and Biodiesel, which are the most commonly used fuels in motor vehicles. Road traffic emissions depend on many factors, such as the type of vehicle (passenger cars, light duty vehicles, heavy duty vehicles, mopeds and motorcycles), the speed at which they travel, the distance travelled, the type of fuel, the engine displacement and weight, the age of the vehicle and the technology it uses for the reduction of NO_x_. In order to calculate the emissions for the study, it was considered that routes involving urban roads have an average speed of 30 km/h, secondary roads of 60 km/h, and that on main roads all vehicles travel at 120 km/h.

The main air pollutants analysed included NO_x_, SO_x_, O_3_, CO, NH_3_ and VOC. The emission of suspended particles (PM) which have a demonstrable impact on health was also calculated [24]. Particles smaller than 30 μm in diameter have an impact on the nose and throat, while those less than 10 μm, such as SO_2_, NO_2_ and ozone, have an impact on the trachea, bronchi and bronchioles. For these calculations, emission factors have been used according to the number of licensed vehicles in Catalonia in 2012 and the 2013 guide to calculating emissions of atmospheric pollutants [25]. Said guide is based on the COPERT 4 v10.0 software program which includes the emission factors described in the European Monitoring and Evaluation Programme (EMEP)/European Environment Agency (EEA) air pollutant emission inventory guidebook [26]. COPERT 4 v10.0 is a tool developed by Aristotle University of Thessaloniki and funded by the European Environment Agency [27]. 

The emission factor used in the study was an average emission factor which takes into account traffic for all types of road and is expressed in g/km. The calculation of emissions was carried out using the formula E = M × N × EF; where E is the emission of the pollutant (g), N is the number of vehicles, M is the distance travelled by the vehicle (km) and EF is the emission factor (g/km). The emission of air pollutants per km is shown in Table 1.

It was considered that an ultrasound scan conducted in a PCC avoided a face-to-face consultation with the radiology service when no subsequent face-to-face visit was made to the service in relation to the same type of ultrasound test in the three months following the ultrasound test conducted by the health centre [6,28].

Although the study focuses on the analysis of the environmental impact derived from the direct displacement of patients, to have a more complete picture we have included impact as a consequence of the mobility of professionals. There are multiple players who can participate in the ultrasound diagnostic process. Air pollution is a mixture of different pollutants and we cannot add up the risk for those pollutants. Epidemiological studies usually rely on a single marker of air quality. As in other previous studies [29], we have selected PM_10_ as the marker of air pollution. The population exposure to PM_10_ was represented by an average population-weighted concentration derived from PM10 concentration tables developed by local authorities.

A descriptive analysis of the different types of ultrasound scans conducted by the centres was carried out for the study according to whether the health centres were urban or rural, and by comparing those tests which required subsequent evaluation by the radiology referral service. The differences between centres were analysed based on the average cost per patient journey and on the average quantity of pollutants emitted in undertaking said journey. The data was processed and analysed using Microsoft Excel and SPSS 23.0 (SPSS Inc., Chicago, IL, USA) programs.

## 3. Results

An initial descriptive analysis of the study data shows that in 2019, the eight doctors who performed ultrasound scans in the Primary Care Centres (PCCs) in Osona county carried out a total of 1556 scans (Table 2). Almost half of the tests (48.5%) corresponded to abdominal ultrasound scans, more than a quarter to vesico-renal examinations and the remainder, in order of frequency, corresponded to ultrasound scans of the thyroid, soft tissue, joints and a small number of vascular ultrasounds. The average number of ultrasound scans, per professional, during the study year was 194.5 (42–449), with a standard deviation of up to 117.

Most of the ultrasound scans 1045/1556 (67.2%) were performed by doctors belonging to rural health centres, while 511/1556 (32.8%) were performed by doctors from urban centres. 

The number of ultrasound scans performed according to the type of examination and characteristics of the centre (rural or urban) adjusted for the population served by the centre (population assigned to the centre * 10,000 inhabitants) showed an average of 229.7 tests for every 104 users assigned (SD 115.7) in rural centres and 108.6 (SD 56.4) in urban centres (Table 3). When comparing this rate according to the type of ultrasound scan, abdominal ultrasounds show a mean of 50.5 (SD 21.3) in urban centres vs. 117.2 (SD 27.7) for rural centres (*p* = 0.06).

Of the total number of ultrasound scans performed, a total of 36 cases required a referral to a specialist radiology service for a face-to-face scan. Therefore, the percentage of face-to-face consultations avoided by conducting an ultrasound scan in a PCC was 97.7% (Table 4).

Table 5 shows the distance of the journeys avoided due to conducting ultrasound scans at PCCs and the time and cost savings of the journey in terms of unused fuel. The average round trip distance between a PCC and the radiology referral service was 17.8 km, with an average saving in travel time of 21.4 min. The journeys avoided during the year the study was conducted totaled 27,123 km, with a total saving of travel time of more than 22 days. Likewise, this meant a saving of 1872 L of fuel, with an associated total cost of €2658 per year.

If we focus on the health professionals’ movements, the variations are minimal or non-existent since neither family doctors nor technicians or radiologists from specialized units changed their mobility. This was different in the case of maintenance staff in charge of installing, updating and maintaining the ultrasound scanners. The maintenance contract included the installation and comprehensive maintenance of the equipment (including those located in the specialized service and those located in primary care centres, as well as at least two preventive visits per year). Car journeys due to the maintenance of the devices accumulates a total of 10 annual trips in the case of ultrasound scanners located in primary care teams, and only two in the case of ultrasound scanners located in the specialized service. We do not have data relating to the travel costs to install and configure the devices for the first use, but this does not appear to be significant as they only involved one visit. Although it seems that the most used equipment requires the greatest amount of technical assistance, there is no evidence that more trips were needed during the year of the study.

Table 6 shows a breakdown of the reduction in air pollutant emissions. The total reduction in pollutants was equivalent to 92.7 kg of carbon monoxide, 22.3 kg of nitric oxide and 4 kg of sulphur dioxide per year. This represents an average reduction on each trip of 61.4 g of carbon monoxide, 14.8 g of nitric oxide and 2.7 g of sulphur dioxide.

As has already been observed when examining the fuel consumption and costs of the journeys avoided, as expected the reduction in pollutant emissions was also significantly higher (for all pollutants studied) in the journeys avoided from rural areas versus those originating from urban areas, given their greater average distance from the radiology referral service.

Demographic mortality data of the year 2019 was obtained from Catalonian Demographical Institute’s (IDCAT) data, and the relative risk estimates (and 95% confidence intervals) for a 10 μg/m^3^ PM10 increase were obtained from previous studies [31]. With 1426 deaths in 2019 from a total population of 153,000 inhabitants, by calculating the impact of reducing the average PM10 concentrations observed in the area (24.52 μg/m^3^) to 20 μg/m^3^ (recommended by WHO) and multiplying with the adjusted relative risk of 1.006 (1.004, 1.008) [30], we determined that reducing PM10 by an average of 4.52 μg/m^3^ could avoid four (3–6) deaths per year. The study shows that the total amount of PM10 generated with the avoided journeys was only 0.00025 ug/m^3^ per day which is not enough to prevent any deaths.

## 4. Discussion

This study analyses the impact that performing ultrasound scans in primary care centres has in terms of avoided journeys to specialized services, time saved, reduced fuel consumption and the consequent decrease in the emission of atmospheric pollutants. The rural and dispersed nature of most of the study centres highlights a greater effectiveness of the intervention in this field. The county in which the study was conducted has a population of 158,334 (160,464 registered residents), of which 54.89% are assigned to a rural health area with under 10,000 inhabitants and less than 150 inhabitants/km^2^. From the patient’s point of view, avoiding an unnecessary journey to the radiology service means, in addition to saving time, a reduction in fuel costs, while the same examination is performed in a manner which is similar to their own GP (primary care level).

This study joins others which show that when PCCs provide tests or services which until now have been offered exclusively by specialized units, it leads to a reduction in patient journeys and thus contributes to a significant reduction in the associated costs for users [32,33] in addition to a reduction in the emission of atmospheric pollutants [6,34]. According to data from the World Health Organization (WHO), air pollution in cities and rural areas is responsible for 4.2 million premature deaths per year due to exposure to particulate matter (PM) which causes cardiovascular and respiratory diseases and cancers [35]. Therefore, the reduction in emissions associated with the journeys shown to have been avoided would have a beneficial effect on the health of the population. In fact, the WHO has recently determined the environmental burden of diseases in each country based on selected risk factors including air pollution, and in the case of Spain the air pollution burden has been estimated to stand at 5800 deaths per year. This calculation assumes a reduction in the average urban levels of PM10 from 30 g/m^3^ to 20 g/m^3^, the average value of PM_10_ in the WHO’s recent recommendations [36].

All factors in this study have been based on the mandatory reporting factors described in the EMEP/EEA air pollutant emissions inventory of the European Environment Agency [37]. Although carbon dioxide is a major factor in environmental pollution, it is not included in the inventory and has not been taken into account in the study as its inclusion could have altered reliability with respect to the calculations made with other factors that are in the EMEP/EEA inventory.

It should be noted that to facilitate the calculations made in the study and extrapolating from the most frequent case in the setting in which this study was conducted, the transport system used to make the journeys was taken to be a private, small family car with average fuel consumption. This study did not take into account any journeys which may have been made by public transport (very scarce or non-existent in certain rural populations), low or zero emission vehicles (hybrid and electric cars), or highly polluting vehicles (obsolete cars or towing a trailer).

Nonetheless, the present study has certain limitations. One being the fact that it is a retrospective study which does not allow us to obtain any information of interest such as data related to the loss of working hours and wages, the amount of stress related to driving, waiting times or additional costs such as parking charges. None of these costs are reflected in the current study. In addition, this study does not take into account factors which increase the cost of performing ultrasound scans in PCCs, such as the need to purchase equipment, the ongoing training of professionals, the additional time the professional needs to dedicate to conducting examinations or the possible increase in demand for ultrasound scans due to greater access to the test. The POCUS model could also lead to overdiagnosis if its use is not limited to the organs upon which the clinical suspicion that motivates the use is based.

One factor not assessed in the study, but which is of great interest, is the environmental impact generated by equipment wastage and consumable materials that might cause air and land pollution. Although this impact is not analysed in this study, we should also consider it in order to have a complete picture of the environmental impact. According to the purchasing department, the disposable materials relating to ultrasounds include gloves, disinfectant liquid, reel hand paper, a bunk bed, transducer covers and conductive gel. Among these materials, the only ones exclusive to ultrasounds are the conductive gel and the transducer covers (the rest are difficult to impute directly to the use of ultrasounds) [38]. The annual purchase of conductive gel for the radiology service was 713 units compared to 107 in primary care; 7776 transducer covers were purchased in the radiology service compared to 144 in primary care. Although the environmental impact of these wastes may be limited, it seems that there is a greater use of consumables and therefore a greater production of waste in the specialized services.

The study could also have evaluated other factors which affect the use of the POCUS model in primary care which go beyond the factors under investigation. Ultrasound scans performed using this model mean that any potential referrals can be optimised, minimizing uncertainty and ruling out certain diseases due to the equipment’s high diagnostic precision. Ultrasound is an additional tool in the diagnosis process, but its use should be limited to certain clinical situations. Its use in the early detection of diseases prevalent in primary care ought to be appropriately evaluated [8].

## 5. Conclusions

This study confirms that the practice of conducting ultrasound scans in primary care reduces the environmental impact of atmospheric pollutants emitted by vehicles by avoiding the journeys which would have been necessary to carry out the scans by attending a specialized radiology service. The avoided journey results in savings of time and money for the user in addition to the reduction of fuel consumption and the emission of atmospheric pollutants. Other studies ought to analyse the potential impact of expanding the portfolio of primary care services on the potential savings in journeys made and their implications for the patients’ work and personal life.

The increase in the number of POCUS programs, which has started to include the use of portable devices (hand-carried or hand-held ultrasounds) in patients’ homes in rural and low-income settings [39,40,41,42], ought to be seen as a new tool and part of a broader strategy to reduce emissions of air pollutants.

Earth pollution and climate change is a reality. The modern healthcare sector contributes towards this grave phenomenon and, at the same time, it is being affected by it. The present study was thus conducted to identify one of the multiple ways in which the health sector can contribute to prevent climate change. Private car travel is a major source of air pollution and Telemedicine has the potential to minimize it by reducing journeys [43]. Potential air pollutant savings are strongly associated with the number of users and appointments that can be replaced by teleconsultations or point-of-care visits. The benefits will depend on the amount, the distance and type of transportation replaced by those visits. Local health initiatives, as modest they are, could contribute to expand this new model of Green Health. Maybe the results of this study can contribute to extending the POCUS model, increasing its future environmental impact.

## Figures and Tables

**Table 1:** Emission of pollutants specific to private vehicles (per passenger and per km).

Pollutant	Formula	Vehicle EF ^1^ (g/km)
Nitrogen oxides	NO_x_	0.8287
Particles with a diameter of less than 10 µm	PM_10_	0.0397
Particles with a diameter of less than 2.5 µm	PM_2.5_	0.0339
Total particles	PM	0.0265
Carbon monoxide	CO	3.4391
Ammonia	NH_3_	0.0201
Volatile organic compounds	VOC	0.31
Non-methane volatile organic compounds	NMVOC	0.2887
Methane	CH_4_	0.0212
Nitrogen monoxide	NO	0.6666
Nitrogen dioxide	NO_2_	0.1621
Nitrous oxide	N_2_O	0.0058
Sulphur dioxide	SO_2_	0.15

^1^ EF: Emission Factor = Emissions (g)/number of vehicles × distance travelled (km). Source: 2013 guide to calculating emissions of atmospheric pollutants [25].

**Table 2:** Types of ultrasound scans performed by the different primary care professionals according to whether they work in a rural or urban centre.

PCC Prof ^1^ *n* (%)	Abdomen	Joints	Soft Tissue	Thyroid	Vesico-Renal	Vascular	Total
P1	20 (47.6)			5 (11.9)	17 (40.5)		42 (2.7)
P2	102 (51.8)	14 (7.1)	8 (4.1)	13 (6.6)	60 (30.5)		197 (12.7)
P3	119 (43.8)	3 (1.1)		40 (14.7)	110 (40.4)		272 (17.5)
**Urban**	**241 (47.2)**	**17 (3.3)**	**8 (1.6)**	**58 (11.4)**	**187 (36.6)**		**511 (32.8)**
P4	52 (48.1)	2 (1.9)	9 (8.3)	13 (12.0)	32 (29.6)		108 (6.9)
P5	48 (35.0)	22 (16.1)	19 (13.9)	15 (10.9)	33 (24.1)		137 (8.8)
P6	56 (28.0)	27 (13.5)	48 (24.0)	10 (5.0)	56 (28.0)	3 (1.5)	200 (12.9)
P7	76 (50.3)	8 (5.3)	12 (7.9)	5 (3.3)	50 (33.1)		151 (9.7)
P8	282 (62.8)		17 (3.8)	65 (14.5)	85 (18.9)		449 (28.9)
**Rural**	**514 (49.2)**	**59 (5.6)**	**105 (10.0)**	**108 (10.3)**	**256 (24.5)**	**3 (0.3)**	**1045 (67.2)**
**Total**	**755 (48.5)**	**76 (4.9)**	**113 (7.3)**	**166 (10.7)**	**443 (28.5)**	**3 (0.2)**	**1556**

^1^PCC Prof: primary care centre professionals; We show in a bold format the grouped results of rural and urban centers’ professionals.

**Table 3:** Ultrasound scan rate per inhabitant in rural vs. urban centres.

PCC Prof ^1^	Abdomen	Type of Ultrasound Scan × 10^4^ Inhabitants				Vascular	$\bar{X}$ Total
		Joints	Soft Tissue	Thyroid	Vesico-Renal		
**Urban centres**	50.7	3.2	1.4	12.9	40.5		**108.6**
U1	65.7	1.4	0.0	21.3	60.1		148.5
U2	35.6	4.9	2.8	4.5	20.9		68.7
**Rural centres**	117.2	11.7	23.2	22.1	54.9	0.6	**229.7**
R1	138.6	35.2	52.6	25.7	102.1	1.8	356
R2	127.1	0.0	6.3	32.0	38.9		204.3
R3	85.9	0.0	10.7	8.6	23.6		128.9

^1^PCC Prof: primary care centre professionals; We show in a bold format the grouped results of rural and urban centers’ professionals.

**Table 4:** Face-to-face consultations and consultations avoided by conducting ultrasound scans in primary care centres.

Ultrasound	Abdomen	Joints	Soft Tissue	Thyroid	Vesico-Renal	Vascular	Total
Type (n)	755	76	113	166	443	3	1556
2nd radiologist consult (n)	19	1	5	4	7		36
Avoided (%)	97.5	98.7	95.6	97.6	98.4	100	97.7

**Table 5:** Reduction in journeys according to distance, time, fuel and cost.

Type PCT Saving	Average Number of Journeys Avoided *			Total of Journeys Avoided *		
	Rural (IC 95%)	Urban (IC 95%)	Total (IC 95%)	Rural	Urban	Total
Distance (km)	20.0 (19.1–20.8)	13.6 (12.6–14.5)	17.8 (17.2–18.5)	20,297.8	6825.2	27,123
Time (day:hour:min)	24.2 (23.5–24.9)	15.8 (14.7–16.9)	21.4 (20.8–22.1)	17:02:20	05:12:36	22:14:56
Fuel (L)	1.4 (1.3–1.4)	0.9 (0.9–1.0)	1.2 (1.2–1.3)	1400.5	470.9	1871.5
Cost (€)	2.0 (1.9–2.0)	1.3 (1.2–1.4)	1.7 (1.7–1.8)	1989.2	668.9	2658.1

* All nonparametric tests (Mann–Whitney U test) to determine if there were significant differences using rurality as an independent variable were significant (*p* < 0.001). Source: Cost of fuel in Spain [30].

**Table 6:** Reductions in the emissions of pollutant gases.

Pollutant	Average Emissions Per Journey * gr (IC 95%)			Total Emissions * (Total Journeys)		
	Total	U	R	Total	U	R
NO_x_	14.8 (14.3–15.3)	11.2 (10.5–12.0)	16.5 (15.9–17.2)	22,328.8	5491	16,655.8
PM_10_	0.7 (0.7–0.7)	0.5 (0.5–0.6)	0.8 (0.8–0.8)	912.5	223.4	680.1
PM_2.5_	0.6 (0.6–0.6)	0.5 (0.4–0.5)	0.7 (0.6–0.7)	1070.8	264	798.8
PM	0.5 (0.5–0.5)	0.4 (0.3–0.4)	0.5 (0.5–0.6)	713.8	175.9	532.9
CO	61.4 (59.2–63.6)	46.7 (43.4–50.0)	68.6 (65.9–71.4)	92,664.7	22,786.5	69,120.2
NH_3_	0.4 (0.3–0.4)	0.3 (0.3–0.3)	0.4 (0.4–0.4)	541.2	133.2	404
VOC	5.5 (5.3–5.7)	4.2 (3.9–4.5)	6.2 (5.9–6.4)	8353.1	2053.8	6230.3
NMVOC	5.2 (5.0–5.3)	3.9 (3.6–4.2)	5.8 (5.5–6.0)	7778.4	1912.4	5802
CH_4_	0.4 (0.4–0.4)	0.3 (0.3–0.3)	0.4 (0.4–0.4)	571	140.7	426.3
NO	11.9 (11.5–12.3)	9.0 (8.4–9.7)	13.3 (12.8–13.8)	17,961.2	4416.7	13,397.5
NO_2_	2.9 (2.8–3.0)	2.2 (2.0–2.4)	3.2 (3.1–3.4)	4367.6	1074.4	3258.3
N_2_O	0.1 (0.1–0.1)	0.08 (0.07–0.08)	0.12 (0.11–0.12)	156.3	38,4.	116.5
SO_2_	2.7 (2.6–2.8)	2.0 (1.9–2.2)	3.0 (2.9–3.1)	4041.5	993.8	3014.7

*All nonparametric tests (Mann–Whitney U test) to check if there were significant differences using rurality as an independent variable were significant (*p* < 0.001). Emissions are calculated in grams (gr) using formula in Table 1: number of vehicles × distance travelled × emission factor by pollutant.
